# Associations between Individual and Combined Polymorphisms of the TNF and VEGF Genes and the Embryo Implantation Rate in Patients Undergoing *In Vitro* Fertilization (IVF) Programs

**DOI:** 10.1371/journal.pone.0108287

**Published:** 2014-09-23

**Authors:** Radia Boudjenah, Denise Molina-Gomes, Antoine Torre, Florence Boitrelle, Stéphane Taieb, Esther Dos Santos, Robert Wainer, Philippe de Mazancourt, Jacqueline Selva, François Vialard

**Affiliations:** 1 Department of Reproductive Biology, Genetics, Gynaecology and Obstetrics, Poissy Saint Germain Medical Centre, Poissy, France; 2 EA 2493, Versailles Saint Quentin University, Versailles, France; Clermont-Ferrand Univ., France

## Abstract

**Background:**

A multiple pregnancy is now considered to be the most common adverse outcome associated with *in vitro* fertilization (IVF). As a consequence, the identification of women with the best chances of embryo implantation is a challenge in IVF program, in which the objective is to offer elective single-embryo transfer (eSET) without decreasing the pregnancy rate. To date, a range of hormonal and clinical parameters have been used to optimize eSET but none have significant predictive value. This variability could be due to genetic predispositions related to single-nucleotide polymorphisms (SNPs). Here, we assessed the individual and combined impacts of thirteen SNPs that reportedly influence the outcome of in vitro fertilisation (IVF) on the embryo implantation rate for patients undergoing intracytoplasmic sperm injection program (ICSI).

**Materials and Methods:**

A 13 gene polymorphisms: FSHR(Asn680Ser), p53(Arg72Pro), AMH(Ile49Ser), ESR2(+1730G>A), ESR1(−397T>C), BMP15(−9C>G), MTHFR1(677C>T), MTHFR2(1298A>C), HLA-G(−725C>G), VEGF(+405G>C), TNFα(−308A>G), AMHR(−482A>G), PAI-1(4G/5G), multiplex PCR assay was designed to genotype women undergoing ICSI program. We analyzed the total patients population (n = 428) and a subgroup with homogeneous characteristics (n = 112).

**Results:**

Only the VEGF(+405G>C) and TNFα(−308A>G) polymorphisms impacted fertilization, embryo implantation and pregnancy rates. Moreover, the combined VEGF+405.GG and TNFα-308.AG or AA genotype occurred significantly more frequently in women with high implantation potential. In contrast, the VEGF+405.CC and TNFα-308.GG combination was associated with a low implantation rate.

**Conclusion:**

We identified associations between VEGF(+405G>C) and TNFα(−308A>G) polymorphisms (when considered singly or as combinations) and the embryo implantation rate. These associations may be predictive of embryo implantation and could help to define populations in which elective single-embryo transfer should be recommended (or, conversely, ruled out). However, the mechanism underlying the function of these polymorphisms in embryo implantation remains to be determined and the associations observed here must be confirmed in a larger, more heterogeneous cohort.

## Introduction

*In vitro* fertilization (IVF) is a complex, multistep process. Poor control of embryo implantation is still a major obstacle to the achievement of a successful pregnancy. Only about 30% of IVF transfer cycles lead to clinical pregnancies [1]. Low implantation rates have prompted physicians to transfer multiple embryos. However, this strategy increases the likelihood of multiple pregnancy, which constitutes a risk for both the mother and the foetuses [2] and is now considered to be the most common adverse outcome associated with IVF. Indeed, multiple pregnancy increases the risk of preeclampsia, hypertension, preterm birth, preterm rupture of membranes, low birth weight, foetal death and cerebral palsy [2]. It is known that reducing the number of embryos transferred (through elective single-embryo transfer (eSET)) minimizes the incidence of multiple pregnancies. Although several strategies for selecting the right patient population (in terms of fertility criteria) for eSET have been suggested and evaluated, individual patient variability has proven to be a major drawback.

Embryo implantation is a multifactorial event that depends on interplay between the blastocyst and the receptive endometrium. Technical progress has led to improved selection of high-quality embryos, as characterized by (i) the presence of 4 to 5 blastomeres at day 2 after insemination or at least 7 to 10 blastomeres on day 3, (ii) the absence of multinucleated blastomeres, (iii) fewer than 20% of cellular fragments on days 2 and 3 after fertilization and (iv) timing of cleavage events [3]. Furthermore, many strategies have been developed for evaluating endometrial receptivity; these include the histological dating of timed endometrial biopsy tissues [4], the quantitation of endometrial cytokines in uterine flushings [5], plasma prostaglandin-E2 (PGE2) and prostaglandin-F2α levels 24 hours prior to embryo transfer [6], non-invasive ultrasound examination of the endometrium [7], transcriptomic analysis of a timed endometrial biopsy [8], and proteomic analysis of the window of implantation [9].

Separately, none of these markers has significant predictive value. However, predictive performance can be improved by combining these parameters.

Despite these advances in blastocyst and endometrium management, there is still a need to optimise individual embryo transfer protocols and thus reduce the likelihood of implantation failure or multiple pregnancy. An additional approach to this problem is based on the pharmacogenetics of embryo implantation. To date, more than 20 reports have described a variety of genetic variants associated or not with the risk of recurrent implantation failure (RIF) in patients undergoing IVF, comparing alleles frequencies between patients with RIF and control group (Table S1). These risk factors include single nucleotide polymorphisms (SNPs) in the genes for tumour suppressor protein 53 [10]–[12], follicle-stimulating hormone receptor (FSHR) [13], [14], methylenetetrahydrofolate reductase (MTHFR) [15]–[17], human leukocyte antigen-G (HLA-G) [18], [19], vascular endothelial growth factor (VEGF) [20], [21], plasminogen activator inhibitor-1 (PAI) [20], progesterone receptor (PR) [22], [23], and cyclooxygenase-2 (COX-2) [24], serotonin transporter (SERT) and serotonin receptor 1A (5-HT1A) [25]. Moreover, it was recently suggested that the implantation process depends on combinations of several genetic and environmental factors. In support of this hypothesis, an oligo-SNP model (including the p53 Arg^72^Pro, PAI-14G/5G and VEGF+1154 A/G polymorphisms) is reportedly associated with RIF [20].

By using allele-specific polymerase chain reactions, we recently reported, for the first time, that the presence of the TNF-308.A allele was associated with higher implantation, pregnancy and multiple pregnancy rates and a lower miscarriage rate after fresh embryo transfer in patients undergoing IVF for male infertility [26].

In the present study, we used a previously described multiplex PCR [27] to evaluate the individual and combined impacts of 13 gene polymorphisms on embryo implantation rates. Variation frequency of eight of the latter (FSHR(Asn^680^Ser), p53(Arg72Pro), MTHFR1(677C>T), MTHFR2 (1298A>C), HLA-G (−725C>G), VEGF(+405 G>C), TNFα (−308A>G) and PAI-1 (4G/5G) have been previously reported for patients with RIF compared with control.

Our objective was to identify (after genotyping) IVF participants who could benefit from eSET and those in whom eSET should be ruled out because of the high risk of implantation failure.

## Materials and Methods

### Subject population

The present study analysed the associations between the participants' genotype (for 13 genes) on one hand with embryo implantation outcome (together with the ovarian response parameters reported in an earlier study) on the other [27]. Two patient populations were studied: the entire population (comprising 428 women) and a subgroup of 112 patients selected as previously described [26], [27]. The entire population group was selected based on one major criterion: women who required an intracytoplasmic sperm injection (ICSI) procedure for male fertility factors without the contribution of any female factor. Sperm were analyzed according to the World Health Organization guidelines.

More stringent inclusion criteria were used for the selected population group, including a normal karyotype, age under 38 years, serum FSH levels below 10 IU/l on Day 3 of the procedure, severe sperm alterations requiring an ICSI procedure, a unique long agonist desensitization protocol associated with recombinant FSH treatment for ovarian stimulation and Caucasian background. Embryos were cultured for 2 days according to our standard procedures. Day 2 embryo scoring was performed according to Steer's criteria (Steer *et al.*, 1992), including morphological grade and blastomere number. At 2–3 days after oocyte retrieval, endometrium thickness was evaluated and 1–3 embryos were transferred.

The study was approved by an independent ethics committee (the Comité Consultatif de Protection des Personnes dans la Recherche Biomédicale; project reference: 01032) and was performed in accordance with the tenets of the Declaration of Helsinki. All the women provided their prior, written, informed consent to participation.

### Genotyping and allele-specific PCR

Genotyping for the 13 polymorphisms was carried out as described earlier [27]. Briefly, nuclear DNA was extracted from peripheral blood leukocytes. For each of the selected SNPs, primer pairs were designed using Primer3 online software (University of Massachusetts Medical School, U.S.A.) (Table S2). Amplification with a PCR was performed for 24 cycles at an annealing temperature of 65°C [27].

### Statistical analysis

Hardy-Weinberg equilibrium was assessed in a chi-squared test. Statistical analysis was performed in three steps as previously reported [27].

To summarize, for each polymorphism, a univariate analysis was performed (using the Mann–Whitney U test) for age, FSH, LH, E2, and rFSH number units, plasma E2 levels two days before oocyte retrieval, the number of mature oocytes, the number of transferred embryos and the embryo score, based on the presence or absence of the allele types. Fisher's test was used to analyze fertilization, cleavage, embryo implantation and pregnancy rates according to the presence or absence of allele types. An inter-group difference was considered to be statistically significant if two different tests gave a result with p<0.05.

A multivariate analysis was performed for all polymorphisms, and all polymorphisms found to be significant at p≤0.30 after Holm's correction were introduced as covariates into GLM models. Variables with p>0.05 and <0.10 were kept in the final models. Lastly, in order to better identify polymorphism interactions, a multilocus analysis was performed with GLM models. All polymorphisms found to be significant at p≤0.075 in the univariate analysis (without Holm correction) were introduced into the multilocus analysis; this enabled us to decrease the number of independent covariates in 1 the models. Backward selection of variables was performed as above, and variables with p>0.05 and <0.10 were kept in the final models.

## Results

The PCR efficiency ranged between 91.8% and 100% (Table S3). The lowest success rate was observed for the VEGF (rs2010963; +405 G>C) genotype. The 13 polymorphisms were in Hardy-Weinberg equilibrium, with a 1% error interval. The allele frequencies in the total patient population and the selected subset were similar to those quoted on the NCBI database (http://www.ncbi.nlm.nih.gov/snp) [27].

In a univariate analysis, only two of the 13 polymorphisms (VEGF (rs2010963; +405 G>C) and TNFα (rs1800629; −308A>G)) appeared to be significantly associated with differences in the fertilization and/or embryo implantation rates. All six of the other polymorphisms previously reported in the literature as more frequent in a group of patients with recurrent embryo implantation failures (FSHR(Asn^680^Ser), p53(Arg^72^Pro), MTHFR1(677C>T), MTHFR2(1298A>C), HLA-G(−725C>G), PAI-1 (4G/5G)) were not correlated with the fertilization, embryo cleavage, embryo implantation or pregnancy rates in our population (data not shown). The five other polymorphisms (AMH(Ile49Ser), AMHR(−482A>G) ESR2(+1730G>A), ESR1(−397T>C), BMP15(−9C>G), which had been selected for evaluation of their association with the ovarian response in a previous study [27]) were not associated with the embryo implantation or pregnancy rates (data not shown) in our study.

In a multivariate analysis, only the (VEGF +405 G>C and TNFα −308A>G) combination was found to be significantly associated with the embryo implantation and pregnancy rates after controlled ovarian hyperstimulation.

When comparing the various genotypes, there were no statistically significant intergroup differences in the sperm parameters.

### The VEGF polymorphism (rs2010963; +405 G>C)

Given that no differences between VEGF+405.GG and VEGF+405.CG genotypes were observed in either population (Tables S4 and S5), patients with these genotype were pooled and compared with VEGF.CC patients.

Tables 1 and 2 show the data on basal hormonal patterns, ovarian stimulation treatment and response, the amount of exogenous FSH administered, the number of retrieved mature oocytes, cleaved embryo rates and the transferred embryo score as a function of the VEGF+405 polymorphism distribution in the selected subgroup (Table 1) and in the total patient population (Table 2). The VEGF polymorphism wasn't associated with any of these characteristics except the plasma E2 level on day 3, which was significantly lower for the VEGF.CC genotypes in the selected subgroup. This result was not observed in the total patient population.

**Table 1:** VEGF alleles in the selected subgroup: description and ART results.

VEGF genotype		VEGF.GG+GC	VEGF.CC	p value
Patient Number		97	7	
Age	mean ± SD	30.25±2.56	29.14±2.12	NS
Baseline hormone level	FSH (IU/L)	6.44±1.8	6.17±1.73	NS
	LH (IU/L)	4.42±2.10	3.98±1.29	NS
	E2 (IU/L)	46.79±29.46	40.75±14.52	0.040
Ovarian stimulation features	FSHr units - number received	2302±799	2146±803	NS
	Serum E2 level on day 2 before oocyte retrieval	2146±797	2598±921	NS
ART results	Number of mature oocytes	8.2±4.16	9.1±1.95	NS
	Fertilization rate	68.0% (532/785)	48.4%(31/64)	0.002
	Cleavage rate (mean ± SE)	94.5±1.5	92.9±1.9	NS
Implantation results	Embryo number per transfer (mean ± SD)	2.1±0.5	2.0±0.6	NS
	Transferred embryo score	25.1±10.1	20.0±6.6	NS
	Embryo implantation rate	19.6% (41/199)	0% (0/14)	0.058
	Pregnancy rate	32.0% (31/97)	0% (0/7)	0.074
	Multiple pregnancy rate after the transfer of two or more fresh embryos	11.2% (10/89)	0% (0/6)	NS

**Table 2:** VEGF alleles in the total patient population: description and ART results.

VEGF genotype		VEGF.GG+CG	VEGF.CC	p value
	Patient Number	364	29	
Age	mean ± SD	30.05±3.25	29.28±19.78	NS
Baseline hormone levels	FSH (IU/L)	7.28±3.26	7.25±3.01	NS
	LH (IU/L)	4.72±2.31	4.76±2.15	NS
	E2 (IU/L)	46.43±27.16	48.8±25.75	NS
Ovarian stimulation features	FSHr units - number received	2384±2409	2408±1028	NS
	Serum E2 level on day 2 before oocyte retrieval	2157±917	2071±1024	NS
ART results	Number of mature oocytes	7.6±4.1	7.0±3.0	NS
	Fertilization rate	63.4% (1748/2745)	55.4% (107/193)	0.022
	Cleavage rate (mean ± SE)	95.0%	94.0%	NS
Implantation results	Embryo number per transfer (mean ±SD)	2.0±0.8	2.0±0.9	NS
	Transferred embryo score	24.5±9.8	23.0 ± 10.0	NS
	Embryo implantation rate	15.8% (108/685)	3.4% (2/58)	0.011
	Pregnancy rate	24.2% (88/364)	6.9%(2/29)	0.033
	Multiple pregnancy rate after the transfer of two or more fresh embryos	6.9% (20/290)	0%(0/24)	NS

In the selected subgroup, the mean fertilisation rate was found to be significantly higher (p = 0.002) in VEGF.GG+GC patients than in VEGF.CC patients (68.0% and 48.4%, respectively). This difference persisted in the total patient population (p = 0.022), with mean rates of 63.4% and 55.4%, respectively.

Similarly, VEGF.GG+GC patients in the selected subgroup had a higher mean embryo implantation rate than VEGF.CC patients (19.6% and 0%, respectively). In view of the small number of VEGF.CC patients (n = 7), this difference was not statistically significant (p = 0.058). However, a statistically significant difference (p = 0.011) was observed in the total patient population, with mean rates of 15.8% and 3.4% for the VEGF.GG+GC and VEGF.CC groups, respectively.

In the selected subgroup, VEGF.GG+GC patients had a higher pregnancy rate than VEGF.CC patients (with rates of 32.0% and 0%, respectively), although this difference was not statistically significant (p = 0.074). However, the difference was statistically significant (p = 0.033) in the total patient population, with mean rates of 24.2% and 6.9% for VEGF.GG+GC and VEGF.CC patients, respectively.

The VEGF genotype was not significantly associated with the multiple pregnancy rate in either the selected subgroup or the total patient population.

There were no significant intergroup differences in sperm characteristics (data not shown).

Considering the population as a whole, the VEGF.CC genotype's sensitivity and specificity for predicting implantation failure were respectively 8.8% and 98.2%.

### The TNF polymorphism (TNFα: rs1800629; −308 A>G)

TNFα genotyping have been published previously with an allele-specific PCR [26], and similar results were obtained using our multiplex PCR. To summarize our previous data [26], in respectively the selected subgroup and the total population, a significant higher implantation (p = 0.0003 and p = 0.0146) and multiple pregnancy rates (p = 0.0037 and p = 0.0238) was observed for TNF.A allele. Pregnancy (p = 0.0239), miscarriage (p = 0.0364) and take home baby (p = 0.0014) were only impacted in the selected population.

Considering the population as a whole, the TNFα.A allele's sensitivity and specificity for predicting embryo implantation were respectively 31.6% and 78.2%.

### The (TNFα −308A>G/VEGF +405 G>C) combination

Four genotype combinations were evaluated: TNF.AA+AG-VEGF.GG+GC (both of which were positive predictors), TNF.GG-VEGF.GG+GC and TNF.AA+AG-VEGF.CC (a positive and a negative predictor) and TNF.GG-VEGF.CC (both negative predictors). We did not observe any significant differences in any of the study parameters when comparing TNF.GG-VEGF.GG+GC and TNF.AA+AG-VEGF.CC (Table S6) in the entire population. Hence, the data for these genotypes were pooled. There were no patients with a TNF.AA+AG-VEGF.CC genotype in the selected subgroup and so three groups were analyzed.

The data corresponding to these three combinations (on age, basal hormonal pattern, ovarian stimulation treatment and response, the amount of exogenous FSH administered, the number of mature oocytes, cleaved embryo rates and score of transferred embryos) are shown in Table 3 (for the selected subgroup) and Table 4 (for the total patient population). There were no differences between the three groups, other than for plasma levels of E2 and FSH on day 3. In the selected subgroup, the day 3 E2 level was significantly higher (p = 0.003) for the TNF.AA+AG-VEGF.GG+GC genotype combination than for the TNF.GG-VEGF.GG+GC combination. In the total patient population, the FSH level was significantly higher (p = 0.005) for TNF.GG-VEGF.GG+GC and TNF.AA+AG-VEGF.CC than for TNF.AA+AG-VEGF.GG+GC patients.

**Table 3:** TNFα/VEGF alleles in the selected subgroup: description and ART results.

Genotype TNFα/VEGF		TNF.AA+AG- VEGF.GG+GC	TNF.GG VEGF.GG+GC	TNF.GG-VEGF.CC	p value
Patient Number		25	72	7	
Age	mean ± SD	29.96±2.94	30.58±3.58	30.2±2.06	NS
Baseline hormone level	FSH (IU/L)	6.17±1.78	6.53±1.80	6.17±1.73	NS
	LH (IU/L)	4.44±2.46	4.41±1.98	4.0±1.30	NS
	E2 (IU/L)	64.35±41.00^a^	40.30±21.05	40.75±14.52	a = 0.003
Ovarian stimulation features	FSHr units - number received	2148±673	2356±837	2146±803	NS
	Serum E2 level on day 2 before oocyte retrieval	2251±931	2111±752	2599±921	NS
ART results	Number of mature oocytes	7.80±4.2	8.31±4.26	9.14±1.95	NS
	Fertilization rate	64.6%^b^ (126/195)	68.8%^c^ (406/590)	48.4% (31/64)	b = 0.020, c = 0.001
	Cleavage rate (mean ± SE)	94.9%	94.4%	93.0%	NS
Implantation results	Embryo number per transfer (mean ±SD)	1.96±0.54	2.08±0.44	2.00±0.58	NS
	Transferred embryo score	25.16±9.66	25.11±9.65	20.00±6.61	NS
	Embryo implantation rate	38.8%^a^^,^ b (19/49)	14.7% (22/150)	0% (0/14)	a = 0.0003, b = 0.005
	Pregnancy rate	48.0%^a^^,^ b (12/25)	26.4% (19/72)	0% (0/7)	a = 0.046, b = 0.0204
	Multiple pregnancy rate after the transfer of two or more fresh embryos	33.3%^a^ (7/21)	4.4% (3/68)	0% (0/6)	a = 0.0002

a : TNFAA+AG-VEGF GG+GC vs. TNFGG-VEGF GG+GC.

b : TNFAA+AG-VEGF.GG+GC vs. TNFGG-VEGFCC.

c : TNFGG-VEGF GG+GC vs. TNFGG-VEGFCC.

**Table 4:** TNFα/VEGF alleles in the total patient population: description and ART results.

Genotype TNFα/VEGF		TNF.AA+AG-VEGF. GG+GC	TNF.GG VEGF.GG+GC or TNF.AA+AG VEGF.CC	TNF.GG-VEGF.CC	p value
Patient Number n = 393		96	273	24	
Age	mean ± SD	30.68±1.4	30.28±2.59	29.0±21.5	NS
Baseline hormone level	FSH (IU/L)	6.47±1.98^a^	7.56±3.56	7.35±3.23	a = 0.005
	LH (IU/L)	4.78±2.65	4.70±2.17	4.70±2.08	NS
	E2 (IU/L)	50.25±29.25	45.02±26.0	49.30±28.35	NS
Ovarian stimulation features	FSHr units - number received	2433±965	2380±967	2271±922	NS
	Serum E2 level on day 2 before oocyte retrieval	2200±1016	2130±883	2200±1019	NS
ART results	Number of mature oocytes	7.28±3.97	7.65±4.18	7.17±2.89	NS
	Fertilization rate	63.1%^b^ (441/699)	63.8%^c^ (1324/2074)	54.5%(90/165)	b = 0.042c = 0.017
	Cleavage rate (mean±SE)	95.0%	95.0%	94.0%	NS
Embryo implantation results	Embryo number per transfer (mean±SD)	1.82±0.80	1.90±0.75	2.12 ±0.85	NS
	Transferred embryo score	24.3±10.39	24.6±9.71	23.3±9.70	NS
	Embryo implantation rate	21.7%^a^^,^ b (38/175)	13.7%^c^ (71/517)	1.9% (1/51)	a = 0.012, b = 0.001, c = 0.016
	Pregnancy rate	29.2%^b^ (28/96)	22.3%^c^ (61/273)	4.2% (1/24)	b = 0.0105, c = 0.036
	Multiple pregnancy rate after the transfer of 2 or more fresh embryos	13.9%^a^^,^ b (10/72)	4.5% (10/221)	0% (0/21)	a = 0.006, b = 0.071

a : TNFAA+AG -VEGF GG+GC vs. TNF AA+AG -VEGF.CC or TNF-GG-VEGF GG+GC.

b : TNF AA+AG -VEGF.GG+GC vs. TNFGG-VEGFCC.

c : TNF AA+AG -VEGF.CC or TNFGG VEGF GG+GC vs. TNFGG-VEGFCC.

In the selected subgroup, TNF.GG-VEGF.CC patients presented a lower fertilisation rate than the TNF.AA+AG-VEGF.GG+GC and TNF.GG-VEGF.GG+GC patients, with mean values of 48.4%, 64.6% and 68.8%, respectively. This was confirmed in the total patient population, with mean rates of 54.5%, 63.1% and 63.8% respectively.

After embryo transfer, patients in the selected subgroup with a TNF.AA+AG-VEGF.GG+GC genotype had a higher embryo implantation than patients with a negative predictor (the TNF.GG-VEGF.GG+GC genotype (p = 0.0003)) or two negative predictors (i.e. the TNF.GG-VEGF.CC genotype (p = 0.005)), with rates of 38.8%, 14.7% and 0%, respectively. This significant difference was confirmed in the total patient population, with rates of 21.7%, 13.7% and 1.9%, respectively.

Similarly, a higher pregnancy rate was observed for TNF.AA+AG-VEGF.GG+GC genotype in the selected subgroup, with rates of 48.0%, 26.4% and 0% for TNF.AA+AG-VEGF.GG+GC, TNF.GG-VEGF.GG+GC and TNF.GG-VEGF.CC, respectively. This difference was also observed in the total patient population, with rates of 29.2%, 22.3% and 4.2%, respectively.

These findings were consistent with a higher multiple pregnancy rate for TNF.AA+AG-VEGF.GG+GC patients (compared with TNF.GG-VEGF.GG+GC and TNF.GG-VEGF.CC patients) in the selected subgroup (with rates of 33.3%, 4.4% and 0%, respectively) and in the total patient population (with rates of 13.9%, 4.5% and 0%, respectively).

## Discussion

Of the 13 polymorphisms investigated here, only two individual SNPs (VEGF+405 G/C and TNFα-308 G/A) and one SNP combination (VEGF+405/TNFα-308) appeared to be significantly associated with embryo implantation and/or pregnancy rates in an ICSI program. However, no associations were found between the other SNPs on one hand and the fertilization, embryo cleavage, embryo implantation or pregnancy rates on the other.

Patients with a VEGF.CC or TNF-308.GG genotype tended to have a lower embryo implantation rate. A combination of these genotypes was associated with a very low embryo implantation rate. To the best of our knowledge, this is the first study to have shown that the (VEGF+405/TNFα-308) combination modifies the implantation potential.

Considering the population as a whole, the TNFα.A allele had a high specificity (78.2%) for predicting embryo implantation and the VEGF.CC genotype had a high specificity (98.2%) for predicting implantation failure. However, the low sensitivities (31.6% and 8.8% for the TNFα.A allele and the VEGF.CC genotype, respectively) suggest that these markers alone cannot be used to predict embryo implantation (which is undoubtedly a multifactorial process).

The VEGF.CC genotype is associated with reduced gene expression [28]. In theory, this could lead to inadequate angiogenesis and thus lower oocyte quality, lower endometrial vascularisation and lower embryo fertilization and implantation rates (as discussed below). In our study, patients with the VEGF.CC genotype had lower oocyte fertilisation and implantation rates.

Given that pathological male factors might have reduced the fertilization, embryo quality and implantation rates, we analyzed the patients' sperm count, sperm mobility and sperm morphology as a function of genotype. No statistically significant intergroup differences were observed.

Vascular endothelial growth factor (VEGF) is a critical regulator of angiogenesis, which in turn is very important for follicular growth and selection of the dominant follicle [29], [30]. The VEGF is expressed by follicular granulosa and theca lutein cells [31]. The production of angiogenic factors by granulosa cells may help to maintain the vasculature and yield a healthy pre-ovulatory follicle [32], [33]. However, thecal rather than granulosa cells production of angiogenic factors is probably responsible for the follicular vascularisation that enables the follicle to grow and develop [30].

It has been suggested that a highly developed follicular vasculature is required to deliver hormones (and their precursors), oxygen and nutrients. The vasculature could therefore have an important role in the selection and growth of the dominant follicle [29], [30], [34], [35]. Thus, greater vascularity may be a primary determinant of follicular dominance. Conversely, low vascularity may limit the supply of nutrients, substrates and hormones to atretic follicles *in vivo* [30], [36]. Literature data also indicate that an active blood supply is essential for obtaining high-quality oocytes [37]. Our present data are in line with previous reports in which enhanced VEGF expression during the follicular phase was found to increase the number of predominant follicles [35], [38]–[40].

Furthermore, VEGF expression in human endometrium is depressed during the proliferative phase; it increases during the late secretory phase and reaches a peak during ovulation [41]–[43]. Given that a low embryo implantation rate could also be due to a defect in embryo invasion, the latter could lead to abnormal cytotrophoblast invasion and thus be associated with pre-eclampsia, pregnancy loss or intrauterine growth restriction. It is known that the VEGF+405.C allele is more frequent in patients with these conditions [44], [45]. Physiological changes resulting from an alteration in VEGF expression level may explain why VEGF polymorphisms are associated with differences in embryo implantation.

We have previously reported that the TNFα-308 A/G polymorphism was associated with embryo implantation rate [26]. Here, we confirmed this finding using a multiplex PCR assay and also identified an effect when the TNFα-308 A/G polymorphism is combined with the VEGF+405 G/C polymorphism.

Tumour necrosis factor and its two receptors are present in endometrium and placental trophoblasts. The factor has both inflammatory and angiogenic properties and is a potent inducer of new blood vessel growth. It also stimulates the proliferation of endometriotic stromal cells [46]. A G/A SNP in the TNFα promoter has already been linked to idiopathic miscarriage [47], [48]. However, many other researchers have failed to find significant associations in this respect [49]–[57]. In the present study, higher implantation and pregnancy rates were observed in patients with the TNFα.AG genotype, which is reportedly associated with higher TNF expression [58], [59]. Our finding is consistent with a previous report of a significant association between the TNFα concentration in endometrial secretions during the implantation window and the likelihood of a clinical pregnancy [60]. According to recent reports, the balance between pro-and anti-inflammatory cytokines is critical for implantation, placental development and pregnancy outcome. Expression of inflammation-associated T helper 1 (Th1) cytokines (such as TNF) during the pre-implantation and implantation period could favour pregnancy [61]. Moreover, TNFα is an important regulator of trophoblastic matrix metalloproteins (MMPs) (such as MMP2 [62] and MMP9 [62], [63]), which are known to mediate the invasion of an artificial extracellular matrix by trophoblastic cells [64].

Abnormal embryo–endometrial dialogue (resulting in embryo implantation failure) is reportedly related to the over- or under-expression of certain genes [65] and to the presence of polymorphic variants [66]. Our present results suggest that (i) VEGF+405 G/C influences oocyte quality (with low fertilization and implantation rates when the C allele is present) and (ii) TNF-308 influences endometrium receptivity. As discussed above, these two polymorphisms influence different steps in embryo implantation, although VEGF's association with endometrial receptivity needs to be confirmed. We also showed that a combination of polymorphisms was associated with embryo implantation. Taking VEGF.GG+GC and TNF.AA+AG as the reference for adequate implantation, the presence of a homozygous VEGF.CC or TNF.GG allele was associated with a low implantation rate and the presence of both alleles was associated with an even lower rate. The relative effect of the combination of SNPs was greater than that of a single SNP.

Our study of the association between TNF and VEGF polymorphisms and the embryo implantation rate had several limitations. Firstly, there is a lack of functional information on the manner in which polymorphisms in the TNF and VEGF genes affect the embryo implantation rate. Secondly, our study did not measure the patients' plasma VEGF and TNF levels. In the future, studies of larger, more heterogeneous cohorts will be needed to extend our understanding of the impact of TNF and VEGF on embryo implantation. If additional research identifies a definitive, causative role for TNF and VEGF polymorphisms in embryo implantation, the clinician could fine-tune his/her strategy for embryo transfer as a function of the presence of the VEGF+405 and TNFα-308 genotypes. Elective single-embryo transfer should only be performed for patients with VEGF.GG+GC and TNF.AA+AG genotypes. In contrast, eSET should not be performed for patients with VEGF.CC and TNF.GG genotypes.

## Supporting Information

Table S1 **Gene polymorphisms reportedly involved in embryo implantation.**
(DOC)

Table S2 **Primer design for the selected SNPs.**
(DOC)

Table S3 **PCR efficiency.**
(DOC)

Table S4 **VEGF alleles in the selected subgroup: description and ART results.**
(DOC)

Table S5 **VEGF alleles in the total patient population: description and ART results.**
(DOC)

Table S6 **TNFα/VEGF alleles in the total patient population: description and ART results.**
(DOC)
