# Supplementary material for: Associations between Individual and Combined Polymorphisms of the TNF and VEGF Genes and the Embryo Implantation Rate in Patients Undergoing In Vitro Fertilization (IVF) Programs
Source: PLoS One. 2014 Sep 23;9(9):e108287. doi: 10.1371/journal.pone.0108287 (PMC4172632; doi:10.1371/journal.pone.0108287)
Supplement: Table S1 — Gene polymorphisms reportedly involved in embryo implantation. (DOC) [file pone.0108287.s001.doc]

**Table S1: Gene polymorphisms reportedly involved in embryo implantation.**

| **Gene name** | **Protein name** | **Association with embryo implantation** | |
| --- | --- | --- | --- |
|  |  | **Positive association** | **Negative association** |
| ***TNF*** | Tumour necrosis factor alpha | (10) |  |
| ***P 53*** | tumour suppressor protein | (11, 12) | (13) |
| ***FSHR*** | Follicle-stimulating hormone receptor | (14, 15) |  |
| ***MTHFR1 and 2*** | Methylenetetrahydrofolate reductase | (16, 17) | (18) |
| ***HLA-G*** | Major histocompatibility complex class I, G | (20) |  |
| ***VEGF*** | Vascular endothelial growth factor | (21, 22) |  |
| ***PAI-1*** | Plasminogen activator inhibitor-1 | (21) |  |
| **PRG** | Progesterone receptor | (19, 23) |  |
| **COX-2** | Cyclooxygenase-2 | (24) |  |
| **SERT 5 and HT1A** | Serotonin transporter and serotonin receptor 1A | (25) |  |
