# Supplementary material for: Associations between Individual and Combined Polymorphisms of the TNF and VEGF Genes and the Embryo Implantation Rate in Patients Undergoing In Vitro Fertilization (IVF) Programs
Source: PLoS One. 2014 Sep 23;9(9):e108287. doi: 10.1371/journal.pone.0108287 (PMC4172632; doi:10.1371/journal.pone.0108287)
Supplement: Table S2 — Primer design for the selected SNPs. (DOC) [file pone.0108287.s002.doc]

Table S2: Primer design for the selected SNPs

| **Gene name** | **Reference sequence** | **Primer (5’-3’)** | **PCR product size** |
| --- | --- | --- | --- |
| AMH | rs10407022 | F1 :CACAGAGGCTCTTGTGGG**C-FAM**  F2 :CACAGAGGCTCTTGTGGG**A**-**HEX**  R : GATAGGGGTCTGTCCTGCAC | 247 |
| AMHR | rs2002555 | F1 :CCTTCCTCTGCCCAAGC**A**-**FAM** F2 : CCTTCCTCTGCCCAAGC**G-HEX**  R : CCAGCTGAGAACCCAGTGAT | 207 |
| BMP15 | rs3810682 | F1 : GAGGAGGACCATCTTGAAAG**G-HEX**  F2 : GAGGAGGACCATCTTGAAAG**C-FAM**  R : ATGAGGCAACTTTGGTCCAG | 197 |
| ESR1 | rs2234693 | F1 :GAGTTCCAAATGTCCCAGC**T-FAM** F2 :GAGTTCCAAATGTCCCAGC**C-HEX**  R : GGGGAAATTGTTTATTGCAAAC | 234 |
| ESR2 | rs4986938 | F1:GGCCCACAGAGGTCACA**G-FAM** GGCCCACAGAGGTCACA**A-HEX**  R : CTTCCTCACACCGACTCCTG | 157 |
| FSHR | rs6166 | F1:GACAAGTATGTAAGTGGAACCA**T**-**HEX**  F2 :GACAAGTATGTAAGTGGAACCA**C-FAM**  R: TGTTTCACCCCATCAACTC | 224 |
| HLA-G |  | F1:TGAAACTTAAGAGCTTTGTGAGTC**C-FAM** F2 :TGAAACTTAAGAGCTTTGTGAGTC**G-HEX** R : AGTTGTGCCTGAGTGCATGA | 191 |
| MTHFR1 | rs1801133 | F1 :GAAGGTGTCTGCGGGAG**C-FAM**  F2 :GAAGGTGTCTGCGGGAG**T**-**HEX** R : AGAACTCAGCGAACTCAGCA | 238 |
| MTHFR2 | rs1801131 | F1 :GAGGAGCTGACCAGTGAAG**C-HEX**  F2 :GAGGAGCTGACCAGTGAAG**A-FAM**   R : ACAGGATGGGGAAGTCACAG | 178 |
| p53 | rs10425222 | F1 :CAGAGGCTGCTCCC**C-FAM** F2 :CAGAGGCTGCTCCC**G-HEX**  R : GACTTGGCTGTCCCAGAATG | 163 |
| PAI-1 | rs1799889 | F :TCA**GGGG**CACAGAGAGAGTC-FAM  R : CAGCCACGTGATTGTCTAGG | 148  149 |
| TNFα | rs1800629 | F1 : ATAGGTTTTGAGGGGCATG**A -FAM**  F2 : ATAGGTTTTGAGGGGCATG**G-HEX**  R : GAGTCTCCGGGTCAGAATGA | 184 |
| VEGF | rs2010963 | F1 :CTCACTTTGCCCCTGTC**G-HEX** F2 :CTCACTTTGCCCCTGTC**C-FAM** R : GAGGCGCAGCGGTTAG | 351 |
