# Supplementary material for: Associations between Individual and Combined Polymorphisms of the TNF and VEGF Genes and the Embryo Implantation Rate in Patients Undergoing In Vitro Fertilization (IVF) Programs
Source: PLoS One. 2014 Sep 23;9(9):e108287. doi: 10.1371/journal.pone.0108287 (PMC4172632; doi:10.1371/journal.pone.0108287)
Supplement: Table S3 — PCR efficiency. (DOC) [file pone.0108287.s003.doc]

**Table S3: PCR efficiency**

| **Gene name** | **Reference sequence** | **PCR efficiency** |
| --- | --- | --- |
| AMH | rs10407022 | 100% (428/428) |
| AMHR | rs2002555 | 100% (428/428) |
| BMP15 | rs3810682 | 100% (428/428) |
| ESR1 | rs2234693 | 98,4% (421/428) |
| ESR2 | rs4986938 | 100% (428/428) |
| FSHR | rs6166 | 99,8% (427/428) |
| HLA-G |  | 99,8% (427/428) |
| MTHFR1 | rs1801133 | 99,3% (425/428) |
| MTHFR2 | rs1801131 | 99,8% (427/428) |
| p53 | rs10425222 | 100% (428/428) |
| PAI-1 | rs1799889 | 100% (428/428) |
| TNFα | rs1800629 | 100% (428/428) |
| VEGF | rs2010963 | 91.8% (393/428) |
