# Supplementary material for: Associations between Individual and Combined Polymorphisms of the TNF and VEGF Genes and the Embryo Implantation Rate in Patients Undergoing In Vitro Fertilization (IVF) Programs
Source: PLoS One. 2014 Sep 23;9(9):e108287. doi: 10.1371/journal.pone.0108287 (PMC4172632; doi:10.1371/journal.pone.0108287)
Supplement: Table S4 — VEGF alleles in the selected subgroup: description and ART results. (DOC) [file pone.0108287.s004.doc]

**Table S4: VEGF alleles in the selected subgroup: description and ART results.**

| VEGF genotype | | VEGF GG | VEGFGC | p |
| --- | --- | --- | --- | --- |
| Patient Number | | 59 | 38 |  |
| Age | mean ± SD | 30.19±3.07 | 30.13±4.27 | NS |
| Baseline hormone level | FSH (IU/L) | 6.50±1.84 | 6.34±1.74 | NS |
|  | LH (IU/L) | 4.28±1.99 | 4.65±2.28 | NS |
|  | E2 (IU/L) | 51.88±32.01 | 35.84±19.55 | NS |
| Ovarian stimulation features | FSHr units - number received | 2271±847 | 2347±728 | NS |
|  | Serum E2 level on day 2 before oocyte retrieval | 2184±856 | 2086±703 | NS |
| ART results | No of oocytes (mean) | 449 | 336 |  |
|  | Fertilization rate | 65%(296/449) | 70%(236/100) | NS |
|  | Cleavage rate (mean ±SE) | 92.3±1.60 | 97.8±1.23 | NS |
| Implantation results | Embryo number per transfer (mean ± SD) | 2.0±0.43 | 2.2±0.49 | NS |
|  | Transferred embryo score | 24.21±10.5 | 26.50±10.08 | NS |
|  | Embryo implantation rate | 21.4%(25/117) | 19.5%(16/82) | NS |
|  | Pregnancy rate | 30.5%(18/59) | 34.2%(13/38) | NS |
|  | Multiple pregnancy rate after the transfer of 2 or more fresh embryos | 13.2% (7/53 ) | 8.3% (3/36) | NS |
