# Supplementary material for: Associations between Individual and Combined Polymorphisms of the TNF and VEGF Genes and the Embryo Implantation Rate in Patients Undergoing In Vitro Fertilization (IVF) Programs
Source: PLoS One. 2014 Sep 23;9(9):e108287. doi: 10.1371/journal.pone.0108287 (PMC4172632; doi:10.1371/journal.pone.0108287)
Supplement: Table S5 — VEGF alleles in the total patient population: description and ART results. (DOC) [file pone.0108287.s005.doc]

**Table S5: VEGF alleles in the total patient population: description and ART results.**

| VEGF genotype | | VEGF GG | VEGFGC | p value |
| --- | --- | --- | --- | --- |
| Patient Number | | 246 | 118 |  |
| Age | mean ± SD | 31.14±7.57 | 30.97±5.44 | NS |
| Baseline hormone level | FSH (IU/L) | 7.10±2.55 | 7.00±2.21 | NS |
|  | LH (IU/L) | 4.59±2.20 | 5.0±2.51 | NS |
|  | E2 (IU/L) | 48.58±29.43 | 41.81±20.91 | NS |
| Ovarian stimulation features | FSHr units - number received | 2406±1008 | 2337±843 | NS |
|  | Serum E2 level on day 2 before oocyte retrieval | 2168±906 | 2131±942 | NS |
| ART Results | Number of matured oocytes (mean) | 1772 | 973 |  |
|  | Fertilization rate | 63% | 64% | NS |
|  | Cleavage rate (mean ± SE) | 94.0±1.36 | 96.0±1.14 | NS |
| Implantation results | Transfer number | 437 | 248 | NS |
|  | Embryo number per transfer (mean ±SD) | 1.8±0.7 | 2.1±0.8 | NS |
|  | Transferred embryo score | 23.73 ± 9.71 | 26.27 ± 9.97 | NS |
|  | Embryo implantation rate | 15.8% (69/437) | 15.7% (39/248) | NS |
|  | Pregnancy rate | 23.0%(56/243) | 27.1% (32/118) | NS |
