# Supplementary material for: Associations between Individual and Combined Polymorphisms of the TNF and VEGF Genes and the Embryo Implantation Rate in Patients Undergoing In Vitro Fertilization (IVF) Programs
Source: PLoS One. 2014 Sep 23;9(9):e108287. doi: 10.1371/journal.pone.0108287 (PMC4172632; doi:10.1371/journal.pone.0108287)
Supplement: Table S6 — TNFα/VEGF alleles in the total patient population: description and ART results. (DOC) [file pone.0108287.s006.doc]

**Table S6: TNFα/VEGF alleles in the total patient population: description and ART results**

| Genotype TNFα/VEGF | | TNF.AA+AG-VEGF. CC | TNF.GG-VEGF.GG+GC | P value |
| --- | --- | --- | --- | --- |
| Patient Number | | 5 | 268 |  |
| Age | mean ± SD | 30.23±6.9 | 30.59±2.3 | NS |
| Baseline hormone level | FSH (IU/L) | 7.0±2.05 | 7.6±3.6 | NS |
|  | LH (IU/L) | 5.0±2.63 | 4.7±2.2 | NS |
|  | E2 (IU/L) | 47,20±44.95 | 45.0±26.25 | NS |
| Ovarian stimulation features | FSHr units - number received | 3045±1359 | 2365±956 | NS |
|  | Serum E2 level on day 2 before oocyte retrieval | 1560±984 | 2141±880 | NS |
| ART results | Number of mature oocytes | 5.60±3.36 | 7.7±4.2 | NS |
|  | Fertilization rate | 60.7% (17/28) | 64% (1037/2046) | NS |
|  | Cleavage rate (mean± SE) | 94% | 95% | NS |
| Implantation results | Embryo number per transfer (mean ±SD) | 1.4±0.89 | 1.9±0.74 | NS |
|  | Transferred embryo score | 21.0±12.8 | 25,66±9.7 | NS |
|  | Embryo implantation rate | 14.3% (1/7) | 13.7% (70/510) | NS |
|  | Pregnancy rate | 20.0% (1/5) | 22.4% (60/268) | NS |
|  | Multiple pregnancy rate after the transfer of 2 or more fresh embryos | 0% | 4.6% (10/218) | NS |
